# Validation of the treatment identification strategy of the HEDIS addiction quality measures: concordance with medical record review

**DOI:** 10.1186/1472-6963-11-73

**Published:** 2011-04-11

**Authors:** Alex HS Harris, Rachelle N Reeder, Laura S Ellerbe, Thomas R Bowe

**Affiliations:** 1 Center for Health Care Evaluation (MPD:152), VA Palo Alto Health Care System, 795 Willow Road, Menlo Park, CA 94025. USA

## Abstract

**Background:**

Strategies to accurately identify the occurrence of specific health care events in administrative data is central to many quality improvement and research efforts. Many health care quality measures have treatment identification strategies based on diagnosis and procedure codes - an approach that is inexpensive and feasible but usually of unknown validity. In this study, we examined if the diagnosis/procedure code combinations used in the 2006 HEDIS Initiation and Engagement quality measures to identify instances of addiction treatment have high concordance with documentation of addiction treatment in clinical progress notes.

**Methods:**

Four type of records were randomly sampled from VHA electronic medical data: (a) Outpatient records from a substance use disorder (SUD) specialty clinic with a HEDIS-qualified substance use disorder (SUD) diagnosis/CPT code combination (n = 700), (b) Outpatient records from a non-SUD setting with a HEDIS-qualified SUD diagnosis/CPT code combination (n = 592), (c) Specialty SUD Inpatient/residential records that included a SUD diagnosis (n = 700), and (d) Non-SUD specialty Inpatient/residential records that included a SUD diagnosis (n = 700). Clinical progress notes for the sampled records were extracted and two raters classified each as documenting or not documenting addiction treatment. Rates of concordance between the HEDIS addiction treatment identification strategy and the raters' judgments were calculated for each record type.

**Results:**

Within SUD outpatient clinics and SUD inpatient specialty units, 92% and 98% of sampled records had chart evidence of addiction treatment. Of outpatient encounters with a qualifying diagnosis/procedure code combination outside of SUD clinics, 63% had chart evidence of addiction treatment. Within non-SUD specialty inpatient units, only 46% of sampled records had chart evidence of addiction treatment.

**Conclusions:**

For records generated in SUD specialty settings, the HEDIS strategy of identifying SUD treatment with diagnosis and procedure codes has a high concordance with chart review. The concordance rate outside of SUD specialty settings is much lower and highly variable between facilities. Therefore, some patients may be counted as meeting the 2006 HEDIS Initiation and Engagement criteria without having received the specified amount (or any) addiction treatment.

## Background

The ability to accurately identify the occurrence of specific health care events, such as episodes of behavioral health treatment, is central to many quality improvement and research efforts. Is the patient who screens positive for alcohol misuse given a brief intervention or referred to specialty addiction treatment? Does a patient discharged from inpatient detoxification receive appropriate outpatient follow-up? Is the patient with alcohol dependence offered pharmacotherapy? Answering each of these questions depends on having a valid method for judging if targeted patients receive the indicated treatment in specific health care encounters. When the scope of these questions involves hundreds of thousands of patients and perhaps millions of encounters, as in system-wide or national quality monitoring efforts, it is important to operationalize the targeted patients and treatments in a way that minimizes labor intensive strategies, such as direct observation or chart review, and maximizes the use of commonly available, pre-existing, and easily accessible data.

As the most widely used set of quality measures in the U.S. managed healthcare industry, the Healthcare Effectiveness Data and Information Set (HEDIS) operationalizes quality measures across many domains of treatment [1], including two quality measures related to addiction treatment - Initiation and Engagement. Initiation and Engagement are intended to measure early involvement in addiction treatment. The treatment identification strategy used in the Initiation and Engagement measures, based on commonly available procedure and diagnostic codes, is feasible and inexpensive but is of unknown validity. In this context, validity refers to the association between the identification of treatment with a particular strategy (e.g., diagnosis and procedure code combinations) and an often more difficult to obtain "gold standard," such as the direct observation of care or determination of addiction treatment by chart review.

In order to classify an outpatient encounter or inpatient stay as addiction treatment, the specifications of the Initiation and Engagement measures rely on combinations of ICD-9 CM diagnosis codes (primary or non-primary), Common Procedural Terminology (CPT) codes, and/or Universal Billing (UB) Revenue codes. For example, an outpatient visit that contains a CPT code 90804 (individual psychotherapy) with a primary or secondary ICD-9 CM code of 305.0 (alcohol abuse) is assumed to include addiction treatment regardless of the location of that service (e.g., addiction clinic, mental health clinic, primary care) or the presence of other diagnoses that might be the target of the service (e.g., depression). It is unknown to what extent encounters classified as addiction treatment actually involve addiction treatment. If encounters are erroneously being counted as addiction treatment, then some patients may meet the Initiation and Engagement criteria when in fact they have not received any, or at least not the requisite amount of, addiction treatment. Equally important, if the validity of the treatment identification strategy varies by facility or between health care systems, then the overall validity of these quality measures for myriad uses is suspect.

Therefore, the aim of this study was to examine if the diagnosis/procedure code combinations used in the 2006 specifications of the HEDIS Initiation and Engagement quality measures for identifying substance use disorder (SUD) care have high concordance with documentation of addiction treatment in clinical progress notes. Our prior work with these quality measures determined that many of the encounters that qualify as addiction treatment under the HEDIS system occur outside of specialty addiction treatment settings [2,3]. Given other research suggesting that, at least within VHA, very little addiction treatment occurs outside of specialty addiction settings [4], we also examined if the validity of the HEDIS treatment identification strategy is different for health care records generated from addiction treatment programs compared to those generated from other settings.

## Methods

### Data Source and Sampling

The data sources for this study were the fiscal year 2005 (FY05) VHA National Patient Care Database (NPCD) Event and Bed Section files, which contain records of every health care encounter for over 5 million veterans who annually receive care from VHA, and VistAweb, which is an intranet web application of the Veterans Health Information Systems and Technology Architecture (VistA) that enables national chart review [5].

In FY05, there were roughly 330,000 unique VHA patients who had at least one encounter with a SUD diagnosis, 120,000 of whom with encounters in a SUD specialty outpatient or inpatient/residential setting. Over 5 million records qualified as addiction treatment under the 2006 HEDIS criteria ("HEDIS-qualified" encounters). As detailed in the HEDIS technical specifications, outpatient records were defined as "HEDIS-qualified" if they contained both a diagnosis and a CPT code from among those listed in Table 1. Although diagnosis/UB Revenue code combinations can also make an encounter HEDIS-qualified, UB Revenue codes were not considered in this study as they are not available in VHA data. Inpatient records were defined as "HEDIS qualified" if they contained either a primary or a secondary SUD diagnosis from those listed in Table 1. Records were defined as being generated from a SUD specialty setting if they included either a VA Decision Support System (DSS) SUD clinic stop or bed section code (see Table 1). Note that most of the inpatient/residential records were admissions to non-acute residential rehabilitation programs.

**Table 1:** Definitions of SUD Diagnoses, Procedures, and VHA SUD Specialty Locations

Code Type	Codes
SUD Diagnoses (ICD-9 CM)	291-292, 303.00-303.02, 303.90-303.92, 304.00-304.02, 304.10-304.12, 304.20-304.22,304.30-304.32, 304.40-304.42, 304.50-304.52, 304.60-304.62, 304.70-304.72,304.80-304.82, 304.90-304.92, 305.00-305.02, 305.20-305.22, 305.30-305.32,305.40-305.42, 305.50-305.52, 305.60-305.62, 305.70-305.72, 305.80-305.82, 305.90-305.92, 535.3, 571.1

Mental Health Related Procedures (CPT)	90801-90802, 90804-90815, 90826-90829, 90845, 90847, 90849, 90853, 90857, 90862, 90870-90871, 90875-90876, 99201-99205, 99211-99215, 99217-99220, 99241-99245, 99341-99345, 99347-99350, 99384-99387, 99394-99397, 99401-99404, 99420

Clinic Stops (Outpatient)	SUD Individual Session (513) SUD Home Visit (514) SUD/PTSD (519) Intensive SUD Treatment (547) SUD Group Session (560) SUD Telephone (545)

Bed Sections (Inpatient)	SUD Residential Rehabilitation (27) High Intensity SUD Treatment Program (74) SUD Domiciliary (86)

Records were randomly sampled from the National Patient Care Database (NPCD) Event (outpatient) and Bed Section (inpatient and residential care) files stratified by four record types: (a) Outpatient records that had a HEDIS-qualified SUD diagnosis/CPT code combination generated from an addiction specialty clinic (n = 700), (b) Outpatient records that had a HEDIS-qualified SUD diagnosis/CPT code combination generated from a non-addiction specialty setting (n = 592), (c) Inpatient/residential records that included a HEDIS-qualified SUD diagnosis or procedure code generated from an addiction specialty unit (n = 700), and (d) Inpatient/residential records that included a HEDIS-qualified SUD diagnosis or procedure code generated from a non-addiction specialty unit (n = 700). For each record type, a chronological list of all records meeting the criteria was constructed, a numeric vector of the same length was randomly generated, and then the records with the highest random numbers were selected to produce the desired sample size. For record type (b), 700 records were initially sampled but 108 were later eliminated from the analyses because they did not meet the HEDIS criteria.

### Progress Note Extraction

After randomly selecting health care records that met our specifications, we extracted the associated clinical progress notes from VistAweb. Although our interest was in extracting the progress note for the specific qualifying encounter, there is no method for precisely matching records located in VHA administrative data to specific progress notes. Although this process was usually straightforward, it was difficult in some cases to determine which one of several progress notes was the target on a particular day. In these cases, we extracted all of the progress notes on the day of interest. The progress notes were then entered into a secured database to enable coding and analysis and all identifying information was removed.

### Missing Progress Notes

For a surprising number of outpatient records (152 of 1292 sampled; 11.8%), no progress notes were found on the day of the selected record. Progress notes are supposed to be written on the day of the encounter. It is possible to write the note later but attach the note to the day of the encounter in the record. The analytic treatment of the missing progress notes has no wholly satisfying solution. Removing these records from the denominator seems justified as we had no legitimate means in these cases to judge if addiction treatment was provided or not. However, this strategy may bias estimates of treatment provision up or down depending on the unknown rate of treatment provision in these encounters. However, this strategy provides an estimate of the rate of SUD treatment provision in records selected with various administrative codes under the assumption that the rate of SUD care in the records with missing notes is similar to records with observed progress notes.

### Content Analysis and Ratings of Progress Notes

Stemler and others [6,7] provided guidance for developing the rating procedure and conducting content analysis, which relied on a selective reduction process, focusing on key words related to addiction treatment (e.g., ASI, ATP, AUDIT-C, CAT-5, SATP, CAGE, DAP, relapse, rehabilitation, sobriety, AA, addiction, alcohol, cocaine, heroin, naltrexone, disulfiram, antabuse, methadone, detoxification). Although key words were useful, they were insufficient in determining the provision of SUD care. Certain contextual factors disqualified a key word occurrence from a SUD care designation. For example, the following statement would be classified as SUD care: "Positive AUDIT-C. SATP consult placed" -- positive SUD screen and referral to specialized care was considered SUD treatment. However, the following statement would not be considered adequate evidence of SUD treatment: "AUDIT-C was positive." First, the AUDIT-C screen alone is not evidence that SUD care will follow when the screen is positive. Even if this note said, "AUDIT-C was positive. May need substance abuse rehabilitation," it would not meet our criteria, since this statement would need to be accompanied by a documented referral for or additional provision of SUD treatment. Extensive assessment interviews, such as the Addiction Severity Index (ASI), were counted as SUD care because these usually occur as part of the treatment planning and monitoring process rather than to assess need. Guidelines such as these were developed to account for the numerous contextual factors inherent in clinical progress notes. In addition, certain headings in the progress notes proved useful in determining the provision of SUD care. For example, the contents of the "chief complaint," "reason for admission," "admitting diagnosis," "discharge diagnosis," and "assessment/plan" helped pinpoint whether SUD care was provided during an encounter.

Using this system, two raters independently classified each of the selected records as documenting or not documenting the provision of SUD treatment. Every 300 notes, the raters compared and reconciled classifications with the help of a third independent rater to help resolve discrepancies. Additionally, a sample of notes for which agreement existed between the two raters was rated by a third rater as a process quality check and validation. Initial inter-rater reliability exceeded 85% and final inter-rater agreement was 100%. The rate of concordance between the HEDIS SUD treatment identification strategy and the raters' determination of SUD treatment was calculated for each record type.

Records were further classified by specific nature of the treatment provided. The treatment categories were developed through an iterative process of rating and sorting a pilot sample of records. A final code book was developed with the following categories for records with documentation of SUD care: (a) Admission/Discharge/Note from an Inpatient Stay with Documented SUD Treatment, (b) SUD Outpatient Care, (c) Detoxification, (d) SUD Assessment, and (e) Outpatient Care Partially-Related to SUD. (See Table 2 for examples of records that were classified in these categories.) Encounters from non-SUD specialty settings and SUD specialty settings with no documentation of SUD care were also coded by type of treatment (see Tables 3 and 4 respectively). Although this report is focused on the documentation of SUD treatment (Yes/No) rather than on the type of care provided, further details regarding the distribution of records into these SUD treatment categories are available from the authors. The institutional review board of Stanford University and research committee of VA Palo Alto Health Care System approved the study protocol.

**Table 2:** Treatment Categories for Records with Documentation of SUD Care

Treatment Category	Examples
Admission/Discharge Note from an Inpatient Stay with Documented SUD Treatment	Admitted to a drug and alcohol program for the treatment of alcohol dependence
	Admitted to domiciliary for homelessness and scheduled to attend SUD relapse prevention groups
	Admitted for a medical condition (e.g., liver cirrhosis) and referred to SUD-related group and individual therapies

SUD Outpatient Care	Treatment in an addiction setting (e.g., SARRTP) that is not only gambling or smoking-related
	Social services (e.g., housing) provided in a SUD specialty setting
	Patient provided methadone dosage as part of drug treatment program (e.g., Drug and Alcohol Program)

Detoxification	Detoxification is the "chief complaint" or the sole "reason for admission"
	**"**Consent to detox" form signed, signaling plan to actually detox

SUD Assessment	Addiction Severity Index (ASI), if not given for a non-SUD related addiction (e.g., gambling, nicotine)

Outpatient Care Partially-Related to SUD	Positive results on a SUD-related screening (e.g., AUDIT-C, CAGE) and the provider takes further action (e.g., makes a referral or recommendation, advises patient to quit)
	Dual diagnosis treatment (e.g., Seeking Safety) for co-occurring PTSD & SUD
	Treatment of SUD and pain disorder

**Table 3:** Types of Treatment in Non-SUD Specialty Records without Chart Documentation of SUD Care*

Treatment	Outpatient (Non-SUD Specialty) (n = 201)	Inpatient (Non-SUD Specialty) (n = 374)	TOTAL (n = 575)
Medicine (e.g., general, internal, dental)	76 (37.8%)	154 (41.2%)	230 (40.0%)
Non-SUD Mental Health	71 (35.3%)	104 (27.8%)	175 (30.4%)
Withdrawal Precautions or Management, no further action	0 (0.0%)	71 (19.0%)	71 (12.3%)
Negative Screen, no relapse prevention	19 (9.5%)	24 (6.4%)	43 (7.5%)
+Screen, -Response	8 (4.0%)	20 (5.3%)	28 (4.9%)
Medication Management (non-SUD)	12 (6.0%)	0 (0.0%)	12 (2.1%)
Homeless Case Management	6 (3.0%)	0 (0.0%)	6 (1.0%)
Social Work or Services (including vocational)	4 (2.0%)	0 (0.0%)	4 (0.7%)
Phone Call or Letter (scheduling or informational - not telephone care)	1 (0.5%)	1 (0.3%)	2 (0.3%)
Cancellation/No show	1 (0.5%)	0 (0.0%)	1 (0.2%)
Treatment for Gambling	1 (0.5%)	0 (0.0%)	1 (0.2%)
Smoking Cessation / Tobacco Counseling	1 (0.5%)	0 (0.0%)	1 (0.2%)
Recreation or Exercise Therapy	1 (0.5%)	0 (0.0%)	1 (0.2%)

* excludes records with missing progress notes

**Table 4:** Types of Treatment in SUD Specialty Outpatient Records without Chart Documentation of SUD Care

Treatment	Outpatient (SUD Specialty) (n = 48)
Non-SUD Mental Health	12 (25.0%)
Medicine (e.g., general, internal, dental)	8 (16.7%)
Smoking Cessation / Tobacco Counseling	7 (14.6%)
Cancellation/No show	5 (10.4%)
Medication Management (non-SUD)	5 (10.4%)
Treatment Information (e.g., schedule, structure)	4 (8.3%)
Social Work or Services (including vocational)	2 (4.2%)
+Screen, -Response	2 (4.2%)
Phone Call or Letter (scheduling or informational - not telephone care)	1 (2.1%)
Recreation or Exercise Therapy	1 (2.1%)
Homeless Case Management	1 (2.1%)

## Results

Table 5 presents the concordance rates (95% CI) between chart review and the diagnosis/procedure code combinations used in the HEDIS Initiation and Engagement quality measures, as well as the facility range of concordance rates and the range of missing notes.

**Table 5:** Chart Documentation of SUD Treatment by Record Type

Record Type	Total Records Selected	Number (%) with Any Chart Documentation	Number with Chart Documentation of SUD Treatment	% of Documented (95% CI) (VISN % Range)
HEDIS-Qualified from SUD Outpatient Clinic	700	601(85.9%)	553	92.0% (90-94%) (82-100%)

HEDIS-Qualified from Non-SUD Outpatient Clinic	592	539 (91.0%)	338	62.7% (59-67%) (36-85%)

HEDIS-Qualified from SUD Inpatient Unit	700	699 (99.9%)	692	99.0% (98-100%) (95-100%)

HEDIS-Qualified from Non-SUD Inpatient Unit	700	695 (99.3%)	321	46.2 % (42-50%) (18-68%)

About 47% of the over 2.2 million outpatient records generated from addiction specialty clinics had a HEDIS qualified SUD diagnosis/CPT code combination. Of the 700 randomly selected records that met these criteria, 601 (85.9%) had a progress note on the day of care, and 553 (92%) of these were found by chart review to have evidence of SUD treatment. We found that among records with progress notes, the concordance rate for those with a primary SUD diagnosis (92%) was not significantly higher than those with a non-primary SUD diagnosis (91%). The specific diagnosis or CPT code connected with the visit did not affect the association with the chart review determination of SUD care. The types of treatment documented in the 48 records without evidence of SUD care are presented in Table 4. The most common types of treatment were non-SUD mental health treatment, other medical care, and smoking cessation. Concordance rates between the HEDIS-qualified SUD diagnosis/CPT combination and chart review determination of SUD treatment varied substantially by facility (VISN), ranging from 82% to 100%.

Of the 592 records generated from a non-SUD specialty outpatient clinic that had a HEDIS-qualified SUD diagnosis/CPT code combination, 539 (91%) had a progress note on the day of care, of which 338 (63%) were found by chart review to have evidence of SUD treatment. Of the 539 records with progress notes, only 26% had a primary SUD diagnosis which did not affect the concordance with chart review determination of SUD treatment. Concordance rates varied substantially by facility (VISN), ranging from 36% to 85%.

Of the 700 SUD specialty inpatient/residential records that included a HEDIS-qualified SUD diagnosis, only one lacked associated chart documentation and 684 of the remaining 699 (98%) were found to have chart review evidence of SUD care. The concordance rate was no different for records with a primary vs. non-primary SUD diagnosis and did not vary substantially by facility (VISN), ranging from 95% to 100%.

Of the 700 inpatient/residential records generated from a non-addiction specialty unit that had a HEDIS-qualified SUD diagnosis, only 5 lacked associated chart documentation and only 321 of the remaining 695 (46.2%) had chart review evidence of addiction treatment. The concordance rate varied substantially by facility (VISN), ranging from 18% to 68%. Of the 695 records with progress notes, 27% had a primary SUD diagnosis which was significantly associated with the likelihood of chart review evidence of SUD treatment (exp(B) = 5.4, p <.001).

## Discussion

In outpatient and inpatient/residential addiction specialty settings, the HEDIS strategy for identifying addiction treatment, using diagnosis and CPT code combinations, has a high concordance with chart review determination of addiction treatment. This may be considered analogous to the true-positive rate. This study did not investigate whether records which were not classified as involving addiction treatment actually have evidence of such treatment in the progress notes (false-negative rate). However, more than half of the HEDIS-qualifying encounters in a given year occur outside of addiction specialty settings where the concordance with chart review determination of addiction treatment is far lower (high false-positive rate). In non-addiction specialty outpatient settings, only 62.7% of the HEDIS-qualifying encounters had chart review evidence of addiction treatment.

Clearly, there is a need to improve the true-positive rate of the treatment identification strategy for encounters occurring outside of SUD specialty settings. This situation highlights the limitations of using a coding system designed for billing purposes in a quality measurement context. Current efforts are ongoing to design electronic medical record systems with quality measurement applications in mind. Systematic capture of data regarding clinician or clinic specialty and the clinical focus of the encounter would be a major positive step. Perhaps, the inclusion of the more specific H codes in the most recent revision of the HEDIS specifications has improved the true-positive rates in non-SUD specialty settings, but this remains to be studied.

The suboptimal true-positive rate in non-SUD specialty care settings also begs the question: How accurate is accurate enough? With the specifications examined in this study, over one-third of non-SUD specialty care encounters classified as SUD care are false-positives, thereby over-counting encounters in both the denominator and numerators of these measures. But limiting the specifications to encounters that occur in SUD specialty care settings would ignore legitimate SUD care happening in other settings. Furthermore, not all claims-based health care data contains data on the treatment specialty of the providing clinic, as is the case in VHA.

Judgments regarding acceptable levels of false-positives need to be made with a full understanding of the clinical, organizational, and quality measurement context. For example, false-positives in the denominator may cause facilities to be accountable for retaining patients in treatment who never really started SUD treatment. In a system that attaches high-stakes consequences to measured performances, eliminating false-positives may be a higher priority than retaining the non-SUD specialty true-positives that are identified. In another system that is interested in encouraging and monitoring SUD treatment that occurs outside of the specialty clinics, but has not attached high-stakes consequences to the measures, the false-positives may be tolerable in order to get some information about the true-positives. The results of this study can aid in balancing these competing priorities and to help gauge the extent to which these measures should be used for various purposes.

Perhaps of greater concern is the high between-VISN variability of concordance, ranging from 36% to 85%. This implies that some facilities are using these codes in a way that map onto addiction treatment more tightly than other facilities. How this affects the performance ratings of specific facilities is unclear. This problem is even greater for non-addiction inpatient settings where the overall concordance with chart review determination of addiction treatment is 46.2% with a between-VISN range of 18% to 68%. These results raise serious questions about the accuracy of the treatment identification strategy of the 2006 HEDIS Initiation and Engagement measures, especially in systems or facilities that contain non-addiction treatment services, such as an integrated health care system like VHA.

## Limitations

These results and implications need to be interpreted in light of several limitations. First, the progress notes in VistAweb are an imperfect record of the health care encounters. Studies attempting to validate the use of progress notes as indicators of what transpired during a health care encounter have found moderate and variable concordance with direct observation and patient surveys [8,9]. Another study found events reported by a standardized patient were often not reported in the medical record and events not reported by a standardized patient were sometimes documented in the record [10]. Relatedly, if clinicians outside of SUD specialty settings are less likely to document SUD treatment even when it is provided, some of our results might be explained by a "documentation bias" rather than real differences in the validity of the treatment identification strategy between SUD and non-SUD settings.

Another limitation of using progress notes as the "gold standard" determination of SUD care is the variability in the level of detail used to describe outpatient visits. For example, some progress notes contained an abundance of key information for determining SUD care, whereas other progress notes lacked enough detail to make the determination with absolute confidence. Because it was necessary to look at key words (e.g., recovery, addiction) in context, notes lacking detailed information were sometimes difficult to code. In these cases, some arbitration was necessary to carefully consider the note-specific contextual factors and the medical language used. Therefore, though not always entirely satisfying in some cases, consensus among the three raters was the most appropriate way to resolve these issues.

Second, we evaluated the 2006 specification of the HEDIS Initiation and Engagement quality measures. Since that time, the care identification strategy has been modified, most notably by removing DRG codes (not evaluated in this study) and adding H-codes to the list of qualifying procedures. The addition of H-codes is important because many are more disease-specific than the Level 1 CPT codes, and H-codes can be used by the many clinical staff in addiction programs who are not licensed independent providers and are not eligible to use Level 1 CPT codes. Future validation studies should examine the effect of these changes on the overall sensitivity and specificity of the treatment identification strategy. Third, CPT codes may be used differently in VHA compared to sites that use them to bill to third parties. Although we are unaware of such differences, this possibility highlights the risk of generalization of these results outside of VHA. Finally, although a specific encounter may not reflect SUD-specific care, this does not mean the patient never received care. However, the focus of this study is an encounter-level analysis to determine which visits should be counted as SUD care in research and quality measurement applications.

## Conclusions

In order to truly operationalize definitions of quality, quality measure specifications must accurately distinguish target from non-target health care encounters. Assessing the validity of the treatment identification strategy is an underappreciated aspect of quality measure validation. In this study, we determined that the treatment identification strategy of the 2006 HEDIS Initiation and Engagement quality measures of addiction treatment had a reasonable high true-positive rate when the care was provided in an addiction specialty setting. However, HEDIS-qualifying encounters that occurred outside of addiction specialty settings (e.g., mental health or general medical clinics) had much lower and variable true-positive rates. Therefore, at least within the VHA, the interpretation of these measures for between-facility comparisons should be restricted to data generated from addiction programs.

## Competing interests

The authors declare that they have no competing interests.

## Authors' contributions

AH designed the study, conducted analyses, and drafted the manuscript. LE and RR managed the chart data, designed and implemented the chart review procedure and codes, and helped in preparation of the manuscript. TB managed the administrative data, conducted analyses, and helped in preparation of the manuscript. All authors read and approved the final manuscript.

## Pre-publication history

The pre-publication history for this paper can be accessed here:

http://www.biomedcentral.com/1472-6963/11/73/prepub
